# Anti-fibrotic action of pirfenidone in Dupuytren’s disease-derived fibroblasts

**DOI:** 10.1186/s12891-016-1326-y

**Published:** 2016-11-11

**Authors:** Chaoming Zhou, Fang Liu, Phillip H. Gallo, Mark E. Baratz, Sandeep Kathju, Latha Satish

**Affiliations:** 1 Department of Plastic Surgery, University of Pittsburgh, Pittsburgh, PA 15261 USA; 2 Department of Orthopaedic Surgery, University of Pittsburgh, Pittsburgh, PA 15261 USA; 3 McGowan Institute for Regenerative Medicine, University of Pittsburgh, Pittsburgh, PA USA; 4 Department of Plastic Surgery, University of Pittsburgh, 3550 Terrace Street, Scaife Hall, S685.2, Pittsburgh, PA 15261 USA

**Keywords:** Dupuytren’s contracture, Palmar fascia fibrosis, Carpal tunnel, Collagen, Alpha-SMA, Smad2/Smad3, Cell migration, Cell contraction

## Abstract

**Background:**

Dupuytren’s disease (DD) is a complex fibro-proliferative disorder of the hand that is often progressive and eventually can cause contractures of the affected fingers. Transforming growth factor beta (TGF-β_1_) has been implicated as a key stimulator of myofibroblast activity and fascial contraction in DD. Pirfenidone (PFD) is an active small molecule shown to inhibit TGF-β_1_-mediated action in other fibrotic disorders. This study investigates the efficacy of PFD in vitro in inhibiting TGF-β_1_-mediated cellular functions leading to Dupuytren’s fibrosis.

**Methods:**

Fibroblasts harvested from (DD) and carpal tunnel (CT)- tissues were treated with or without TGF-β_1_ and/or PFD and were subjected to cell migration, cell proliferation and cell contraction assays. ELISA; western blots and real time RT-PCR assays were performed to determine the levels of fibronectin; p-Smad2/Smad3; alpha-smooth muscle actin (α-SMA), α2 chain of type I collagen and α1 chain of type III collagen respectively.

**Results:**

Our results show that PFD effectively inhibits TGF-β_1_-induced cell migration, proliferation and cell contractile properties of both CT- and DD-derived fibroblasts. TGF-β_1−_induced α-SMA mRNA and protein levels were inhibited at the higher concentration of PFD (800 μg/ml). Interestingly, TGF-β_1_ induction of type I and type III collagens and fibronectin was inhibited by PFD in both CT- and DD- derived fibroblasts, but the effect was more prominent in DD cells. PFD down-regulated TGF-β_1_-induced phosphorylation of Smad2/Smad3, a key factor in the TGF-β_1_ signaling pathway.

**Conclusion:**

Taken together these results suggest the PFD can potentially prevent TGF-β_1−_induced fibroblast to myofibroblast transformation and inhibit ECM production mainly Type I- and Type III- collagen and fibronectin in DD-derived fibroblasts. Further in-vivo studies with PFD may lead to a novel therapeutic application in preventing the progression or recurrence of Dupuytren’s disease.

## Background

Dupuytren’s disease (DD) is a common fibroproliferative disorder of the hand that is often progressive and eventually can cause contractures of the affected fingers [1]. DD is considered to be caused by a defect in the process of wound healing or by an excessive or abnormal response to wounding [2]. DD is a multifactorial and complex disease, and has been reported to have an association with inherited genetic markers, alcohol and tobacco use [3], and different systemic diseases such as diabetes [4], and epilepsy [5]. The disease usually begins with the formation of highly vascularized nodules and over the years develops into collagenous rich cords. Myofibroblasts that share characteristic features of both fibroblasts and smooth muscle cells have been shown to play a critical role in tissue contraction of DD [6, 7]. Acting along with this cell population are several growth factors and cytokines that are implicated in the progression of the disease [2, 8].

Dupuytren’s disease commonly affects populations of northern European descent, with the prevalence of DD in the general population of Western countries ranging from 0.6 to 31.6 % [9]. The prevalence rate in the United States is reported to be >7 % [10]. In the USA, disability and treatments for DD account for significant losses to the economy and substantial direct and indirect costs [11]. At present there is no cure for DD. The cornerstone of treatment has, historically, been surgical excision of involved palmar fascia release of diseased skin. More recently percutaneous needle fasciotomy and collagenase injections in the cords, is practiced as an alternative to more invasive surgery [12, 13]. Recurrence rates seem to be higher with the less invasive techniques and these strategies do have their own unique complications [14, 15]. Recurrence of DD overall ranges from 8 to 66 % depending on the severity and treatment of disease [16], underscoring the importance of additional research on the causes and factors related to recurrence.

Among all the growth factors that have been studied in DD, transforming growth factor – β (TGF-β) is shown to play a major role in the pathophysiology of DD [17, 18]. In fibroblasts derived from Dupuytren’s affected and unaffected tissue, TGF-β can upregulate α-SMA and is recognized to be the key inducer in differentiating a quiescent fibroblast to a contracting myofibroblast [18]. The inhibition or reversion of the myofibroblast phenotype to normal may mitigate disease progression and severity. Previous studies from our laboratory have shown that increasing cyclic AMP (cAMP) levels via forskolin (an adenylate cyclase (AC) activator) has potential to inhibit TGF-β-induced myofibroblast formation and accumulation of ECM components in DD-derived fibroblasts [19]. Overproduction of cAMP that can provide a means to blunt fibrosis has also been shown in cardiac and pulmonary fibroblasts [20, 21]. Clinical data about the usage of forskolin are not yet available as the risk-benefit ratio is not fully evaluated [22].

In the present study, we have chosen to investigate the action of pirfenidone (PFD; 5-methyl-1-phenyl-2(1H)-pyridone) a small molecule well-documented for its antifibrotic and anti-inflammatory properties in a variety of pre-clinical and in vitro models in different organs, including fibrosis of the lung [23], kidney [24], heart [25], liver [26], and skin [27]. PFD has been shown to inhibit both production and activity of TGF-β1, a cytokine that stimulates collagen synthesis and inhibits its degradation [28], and also to reduce the production of other fibrogenesis mediators, such as fibronectin and connective tissue growth factor (CTGF) [29, 30]. The ability of PFD to combat the fibrosis seen in DD has not previously been reported. PFD is an FDA approved drug for the treatment of idiopathic pulmonary fibrosis in the United States, and thus represents an attractive potential therapy for Dupuytren’s disease.

In this study we examine the ability of PFD to modulate TGF-β_1_-mediated actions on the DD fibroblast’s functional properties, namely cell proliferation, cell migration and cell contraction. In addition, we also examine the effect of PFD on the canonical signaling pathway known to mediate TGF-β_1_ effects in other fibrotic systems.

## Methods

### Cell culture

Primary cultures of fibroblasts were isolated from freshly resected DD cord tissue and normal palmar fascia (CT cells) as previously described [31]. DD cord and CT fascial tissue samples were surgically resected at the Division of Upper Extremity Surgery, Department of Orthopaedic Surgery, Allegheny General Hospital, Pittsburgh, PA. All subjects signed the written informed consent forms, and the protocol was approved by the Allegheny-Singer Research Institute’s Institutional Review Board (IRB protocol no. RC-4040). The study protocol strictly conformed to the ethical guidelines of the 1975 Declaration of Helsinki. Cells are maintained in alpha-MEM medium (Invitrogen™, ThermoFisher Scientific, Pittsburgh, PA) with 10 % FBS and Penicillin/Streptomycin antibiotics (Gibco®, ThermoFisher Scientific) with 5 % CO_2_. Cells used for all experiments were within passages two to seven.

### Cell viability/proliferation

CT- and DD-cord-derived fibroblasts (5 × 10^4^) seeded on a 24 well plate were grown overnight in alpha-MEM medium containing 10 % FBS. Following day, cells were switched to alpha-MEM medium containing 0.1 % dialyzed FBS for 24 h followed by treatment with different concentrations of PFD (0, 200, 400 and 800 μg/mL) with or without 10 ng/ml TGF-β1 in low-serum medium for additional 24 h. After the incubation period, cells were photographed, and MTT assay was performed using the CellTiter 96® Non-Radioactive Cell Proliferation Assay kit obtained from Promega Corporation (Madison, WI) [31]. Cells were replaced with fresh growth medium and dye solution and incubated at 37 °C for 2 h. After adding solubilization/stop solution, cells were incubated for another 1.5 h at 37 °C. Results were obtained using a plate reader by measuring absorption at 570 nm of 100 μl aliquots placed into a Corning 96 well flat transparent plate.

### Lactate Dehydrogenase (LDH) cytotoxicity assay

LDH is a cytosolic enzyme that is not normally discharged outside of the cell, but is released into the cell culture medium upon damage to the cellular membrane. Measurement of extracellular LDH release into the culture medium can be used to assay cellular toxicity. CT- and DD-derived fibroblasts (5 × 10^4^) were seeded in a 24 well plate and were grown overnight in alpha-MEM medium containing 10 % FBS. Following day, cells were switched to alpha-MEM medium containing 0.1 % dialyzed FBS for 24 h followed by treatment with different concentrations of PFD (0, 200, 400 and 800 μg/mL) in low-serum medium for an additional 24 h. After 24 h of exposure, extracellular LDH activity was measured using the In Vitro Toxicology Assay Kit, Lactate Dehydrogenase-based (Sigma-Aldrich, St. Louis, MO) according to the manufacturer’s instructions. The absorbance of LDH release (from medium) was separated and measured using a plate reader at a wavelength 490 nm (primary) and 690 nm (background) by aliquoting 30 μL into a Corning 96 well flat transparent plate. LDH activity was expressed as fold change versus control samples after the background subtraction.

### Cell migration

Cell migration assays were done using the Oris™ Cell Migration assay (Platypus Technologies, Fitchburg, WI) which offers an alternative to the “scratch” test which can vary between experiments and can disrupt extracellular matrix which is vital for cell migration. In brief, this procedure employs a stopper dissolvable biocompatible gel (BCG) to create the cell-free gap in the center of a well in a microplate. Cells seeded into each well are initially restricted from adhering to the center of each well creating a “detection zone”. CT- and DD-cord derived fibroblasts were stained with 3 μM CellTracker™ Red CMTPX Dye (Molecular Probes™, ThermoFisher Scientific) in alpha-MEM medium containing 10 % FBS for 30 min. Cells (1 × 10^4^) were seeded into the wells containing Oris™ Cell Stoppers in Fibronectin Coated or Non-Coated 96- well plate (Platypus Technologies, Fitchburg, WI) and incubated overnight. Following day, the cell stoppers were removed carefully using the Stopper tool leaving the internal controls. Cells were quiesced in the medium containing 0.1 % dialyzed FBS for 24 h. Cells were treated with PFD (800 μg/mL) with or without 10 ng/ml TGF-β_1_ in low-serum medium for additional 24 h. The stoppers in the internal control well were removed just before imaging. The cell migration was assessed by a number of cells that enter the detection zone using NIH Image J 1.44p, available in the public domain at http://imagej.nih.go/ij.

### Stressed Fibroblast -Populated Collagen Lattice Assay (sFPCL)

Cell contractility of the fibroblasts was tested using FPCL assay as previously described with slight modifications [32]. In brief, collagen lattices were prepared by mixing cell suspensions with a neutralized solution of type I collagen matrix (four parts rat tail collagen, one part neutralization solution; two parts 0.34 M NaOH: 3 parts 10X Waymouth media). Cells were exposed to alpha-MEM medium containing 0.1 % dialyzed FBS 24 h prior to plating in 24-well plate. Each well in a 24-well culture dish contained 1 × 10^5^ of CT- and DD-derived fibroblasts in 0.5 ml of the collagen mixture along with or without PFD (800 μg/ml) and TGF-β_1_ (10 ng/ml). Following FPCL polymerization (1 h, 37 °C), 0.5 ml of alpha-MEM medium containing 2 % FBS was added to the top of each lattice and incubated overnight. After 24 h incubation, the attached FPCLs were then manually released from the sides of the culture plates and digitally scanned every 24 h after release until day 6. The area of the lattices was quantified using Adobe Photoshop and was plotted.

### Quantitative Real-time RT-PCR (qRT-PCR)

Total RNA isolated (RNeasy Micro Kit, Qiagen Inc, Valencia, CA) from CT- and DD-derived fibroblasts treated and untreated with PFD (800 μg/ml) and TGF-β_1_ (10 ng/ml) was subjected to real-time RT-PCR to determine the mRNA expression levels for α-SMA (*ACTA2*), type I collagen (*COL1A2*), and type III collagen (*COL3A1*). Human *GAPDH* was used as an normal endogenous control. Real-time RT-PCR was performed using kits obtained from Applied Biosystems (Applied Biosystems®, ThermoFisher Scientific) that utilize FAM™Taqman®MGB probes and a Taqman®Universal PCR Master Mix. 100 ng of total RNA from samples were used for the reverse transcriptase (RT) reaction along with random primers (100 ng) and with M-MLV reverse transcriptase (Invitrogen). Taqman probes for above-noted gene products were purchased from Applied Biosystems. The remaining protocol parameters for subsequent real- time PCR was followed as described previously^19^. Applied Biosystems transcript-specific assays used were *COL1A2* (ID- Hs01028971_ml), *COL3A1* (ID-Hs00943793), *ACTA2* (ID-HS00426835_g1) and *GAPDH* (ID-Hs99999905_m1). Using the comparative critical cycle (Ct) method the expression levels of the target genes were normalized to the *GAPDH* and the relative abundance was calculated. Data were analyzed using the 7900 HT SDS software version 2.2.2 provided by Applied Biosystems.

### Western blotting

CT- and DD-derived fibroblasts (2 × 10^5^) were seeded in six well plates with growth medium containing 10 % FBS overnight followed by exposing the cells in 0.1 % dialyzed FBS-containing medium for 24 h. Cells were treated with 800 μg/ml PFD with or without 10 ng/ml TGF-β_1_ in low-serum medium for an additional 24 h. Following the treatment cells were washed with ice-cold PBS once and then lysed in M-PER™ Mammalian Protein Extraction Reagent (ThermoFisher Scientific) with proteinase inhibitors (cOmplete ULTRA Tablets, Mini, EDTA-free) and Phosphostop purchased from Roche Diagnostics Corporation (Indianapolis, IN). Proteins were resolved by SDS-PAGE, and standard Western blotting procedure was applied. Primary antibodies used were: mouse monoclonal anti-Actin α Smooth Muscle Antibody (predicted and observed molecular weight is 42 kDa 1:5000; Sigma-Aldrich, St. Louis, MO), mouse monoclonal anti-GAPDH antibody, detects a band approximately at ~36 kDa and the predicted molecular weight is 40.2 kDa (1:5000; Abcam, Cambridge, MA), phosphor-Smad2 (Ser465/467)/Smad3(Ser423/425)(D27F4) (1:1000) Rabbit mAb (1:1000) (Cell Signaling, Boston, MA). The predicted molecular weight for pSMAD2 is 60KDa and for pSMAD3 is 52KDa. The observed molecular weights approximate the predicted molecular weights.

Membranes were washed and then incubated for an hour in room temperature with IRDye 800CW Gt Anti-Rabbit IgG (H + L) antibody and IRDye 680LT Anti-Mouse IgG (H + L) antibody (1:15,000) (Li-COR Bioscience, Lincoln, NE). Infrared fluorescence was detected by the Odyssey® Imaging System (Li-COR Bioscience). The protein bands scanned were analyzed using NIH Image J 1.44p. The data obtained was normalized with GAPDH.

### Enzyme- Linked Immunosorbent Assay (ELISA)

CT- and DD-derived fibroblasts (1 × 10^4^) seeded in 96 well plates were grown in growth medium overnight, and cells were changed to medium containing 0.1 % dialyzed FBS for 24 h. Cells were treated with 800 μg/mL of PFD with or without 10 ng/ml TGF-β_1_ in low-serum medium for another 24 h. Following the treatment, secreted proteins in the growth medium were collected in the conditioned medium, which was centrifuged for 10 min to collect the supernatant. Fibronectin levels were analyzed in the supernatants using Human Fibronectin SimpleStep ELISA Kit from Abcam according to the manufacturer’s instructions.

## Results

### Effect of PFD on the proliferative efficiency of CT- and DD-derived fibroblasts

**Fig. 1:**
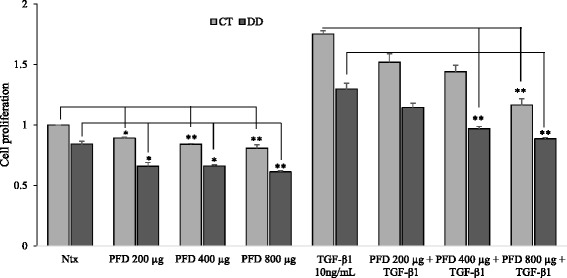
Cell proliferative ability was diminished after the addition of Pirfenidone. (*a*) CT- and (*b*) DD-derived fibroblasts derived from four different patient samples (N = 4/group) were exposed to varying concentrations of PFD (0, 200, 400, 800 μg/ml) and TGF-β_1_ (10 ng/ml). Cells were then subjected to MTT assay to determine the effect of PFD on basal and TGF-β_1_-induced cell proliferation. Cell proliferation is displayed as absorbance values of untreated CT-derived fibroblasts normalized to 1 and compared with other treatments. Data are shown as mean ± SEM of the averages of triplicate reads for each culture derived from the four different patient samples both for CT- and DD-derived fibroblasts. Statistical analysis was performed using one-way ANOVA. *p < 0.05; **p < 0.01

**Fig. 2:**
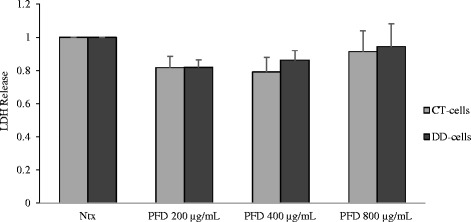
Cell viability was not affected by the presence of Pirfenidone. CT- and DD-derived fibroblasts derived from three different patient samples (N = 3/group) were incubated with different concentrations of PFD (0, 200, 400, 800 μg/ml), and the extracellular LDH release into the culture medium was measured as described in Materials and Methods. Data are shown as mean ± SEM of the averages of triplicate reads for each culture derived from the four different patient samples both for CT- and DD-derived fibroblasts. Ntx = no treatment (neither PFD nor TGF-β_1_ added control). Statistical analysis was performed using one-way ANOVA. *p < 0.05; **p < 0.01

Various concentrations of PFD (0, 200, 400, 800 μg/ml) were tested on CT- and DD-cord derived fibroblasts with or without the stimulation of TGF-β_1_. A dose-dependent effect on cell proliferative efficiency was seen in both CT-and DD-cord derived fibroblasts (Fig. 1a and b). A statistically significant inhibition of both basal and TGF-β_1_ stimulated cell proliferation was observed at all the different concentrations, and highest inhibition was seen at 800 μg/ml. In order to confirm that the reduction of cell proliferative efficiency observed was not due to cytotoxicity we performed LDH assay and determined that there was no cytotoxic effect observed 24 h after treating with different doses of PFD (Fig. 2). No change in cellular morphology (data not shown) was observed, confirming the non-toxic effect of PFD even at higher concentrations on both CT- and DD-cord derived fibroblasts. To determine the functional significance of inhibition of PFD on CT and DD-cord derived fibroblasts, 800 μg/ml of PFD was used for further experimentation.

### PFD significantly inhibits basal and growth factor-induced cell migration on both 2D and 3D surfaces

**Fig. 3:**
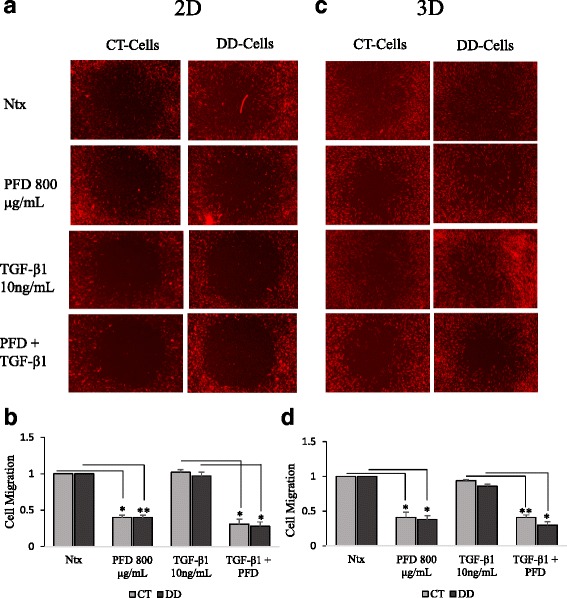
Pirfenidone suppressed basal and TGF-β_1_ induced cell migration. CT- and DD-derived fibroblasts derived from three different pateint samples (N = 3/group) were maintained in low-serum medium for 24 h, then were left untreated (Ntx) or exposed to PFD (800 μg/ml) in the presence or absence of TGF-β_1_ (10 ng/ml) to perform the 2D (**a** & **b**) and OrisTM 3D (**c** & **d**) cell migration assays. Shown here are representative images of three different experiments from three different cultures (N = 3/group) of CT- and DD-derived fibroblasts done for 2D (**a**) and 3D (**c**) migration assays in triplicate. NIH- image analysis was used to assess the number of cells that entered the detection zones after various treatments. Data are shown as mean ± SEM of the averages of triplicate reads for each culture derived from the three different patient samples both for CT- and DD- derived fibroblasts. Statistical analysis was performed using one-way ANOVA. *p < 0.05; **p < 0.01, ***p < 0.001

It is still unclear what role migration of cells plays in the development of the diseased cord in DD disease, although given the severity of contractures that can form some migration of cells as well as cellular contraction seems likely. Our previous studies have shown that the basal cell migratory potential of DD cells was much higher than that of CT- derived fibroblasts (data not shown). In the present study, we investigated the effect of PFD (800 μg/ml) on basal and TGF-β_1_-induced cell migration on fibronectin non-coated (2D) and coated surfaces (3D). No significant cell migratory stimulus was observed from the addition of TGF-β_1_ in both CT-and DD-cord derived fibroblasts in both fibronectin non-coated (Fig. 3a &b) and coated surface plates (Fig. 3c &d). Interestingly, a significant inhibition of basal cell migration was noted in both CT-and DD-cord derived fibroblasts in both 2D and 3D assays when the cells were treated with PFD. The addition of PFD to TGF-β_1_-stimulated cells similarly reduced cell capacity to migrate.

### PFD inhibition of basal- and TGF-β_1_- induced cell contraction is evident in both CT- and DD-cord derived fibroblasts

**Fig. 4:**
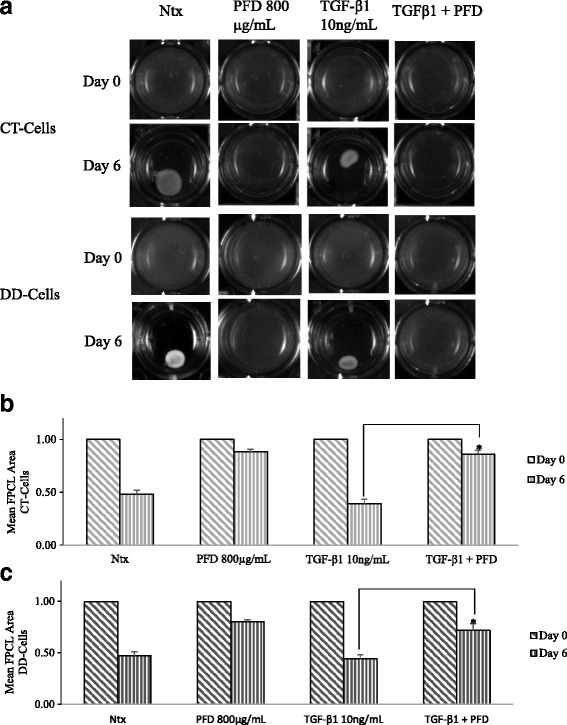
Cellular contractile ability was diminished in the presence of Pirfenidone. CT- and DD-derived fibroblasts derived from four different patient samples (N = 4/group) were left untreated (Ntx) or exposed to PFD (800 μg/ml) in the presence or absence of TGF-β_1_ (10 ng/ml) to perform the stressed fibroblast populated collagen lattice (sFPCL) assay. Collagen lattices cultured for 24 h were detached from the surface of the well, and the digital images of the floating lattices were captured at different time points (day 0- day 6). **a** Shown here are representative images of four independent experiments performed in duplicate. **b** & **c** Data were obtained using Adobe Photoshop to analyze photographic images; data are shown as the area of the contracted collagen lattice normalized to the average area of contraction seen in untreated cells, set as a baseline value of 1. Each data point represents the mean ± SEM of the averages of triplicate reads for each culture derived from the four different patient samples both for CT- and DD-derived fibroblasts. Statistical significance was determined using one-way ANOVA. *p < 0.05; **p < 0.01

**Fig. 5:**
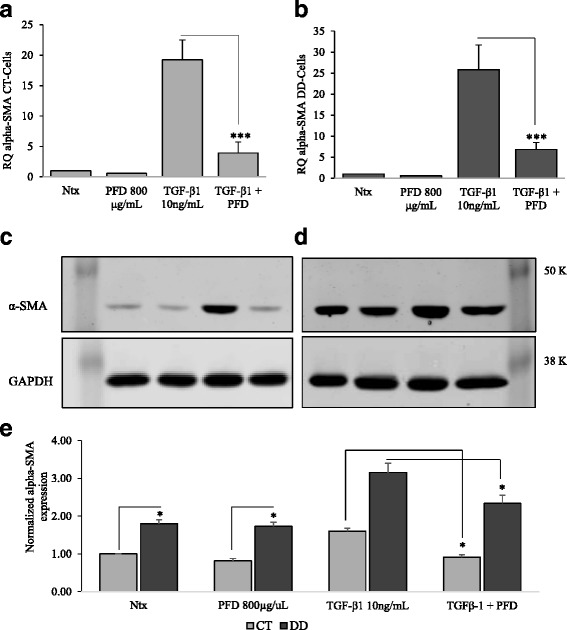
Inhibition of α-SMA levels was evident after addition of Pirfenidone. CT- and DD-derived fibroblasts derived from three different patient samples (N = 3/group) of were left untreated (Ntx) or were stimulated with PFD (800 μg/ml) in the presence or absence of TGF-β_1_ (10 ng/ml) in α-MEM medium containing 0.1 % dialyzed FBS. Twenty-four hours later cell lysates were collected to determine the mRNA and protein expression of α-SMA by real-time RT-PCR (**a** & **b**) and Western blot analyses (**c** & **d**). Real-time RT-PCR experiments were done on three independent cultures of each of the cell types. Values are mean ± SEM of three independent studies performed in triplicate. One-way ANOVA test was used to determine the statistical significance. *p < 0.05, **p < 0.01. Western blot analyses shown here are representative images of experiments performed using three independent primary cultures of CT- and DD-cord derived fibroblasts. Proteins obtained from different patients were processed in parallel to confirm the changes that are observed are consistent. Protein accumulation was quantified by densitometry using GAPDH as a loading control (**e**)

TGF-β_1_ is a growth factor known to induce fibroblast contraction, and it is known to play a critical role in the development of Dupuytren’s disease. Indeed, the deforming force of cellular contraction, on a tissue level, must play a critical role in the development of the extreme contractures that can develop. We determined that TGF-β_1_ induced contraction of both CT-and DD-derived fibroblasts, whereas addition of PFD abrogated both basal- and TGF-β_1_-mediated- contraction (Fig. 4a, b & c). Since α-SMA is the major protein involved in the molecular generation of cellular contraction force, we questioned if the increase in α-SMA levels engendered by TGF-β_1_ can be reduced by the addition of PFD. We found that PFD substantially inhibited TGF-β_1_- induced α-SMA at both mRNA (Fig. 5a & b) and protein levels (Fig. 5c & d) in both CT- and DD-derived fibroblasts. PFD by itself did not have any significant effect on α-SMA mRNA or protein levels in both cell types. These results again strongly indicate that DD fibroblastic cells, in a manner similar to normal fibroblasts, are responsive to PFD action, highlighting the potential of the drug as a candidate to inhibit myofibroblast formation in DD, in turn likely reducing DD contractures.

### PFD attenuates type I and type III collagen subunit mRNA expression in both CT- and DD-derived fibroblasts

**Fig. 6:**
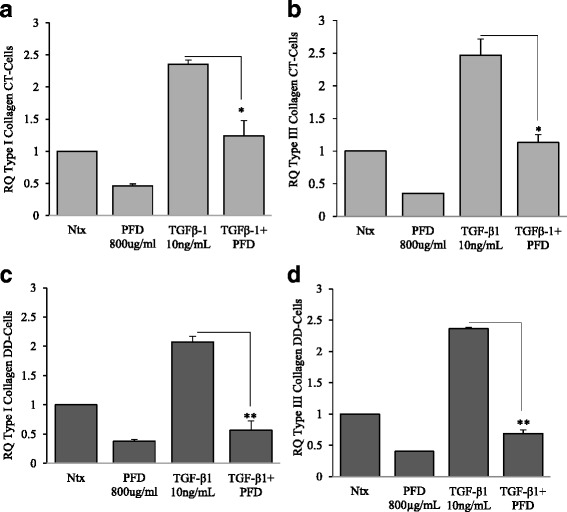
Pirfenidone effectively inhibited TGF-β_1_-induction of α-2 chain of Type I and α-1 chain of Type III collagen. CT- and DD-derived fibroblasts derived from three independent patient samples (N = 3/group) were left untreated (Ntx) or exposed to PFD (800 μg/ml) in the presence or absence of TGF-β_1_ for 24 h. Twenty-four hours later RNA was extracted and mRNA expression levels of type I and type III collagen of CT- (**a** & **b**) and DD-derived fibroblasts (**c** & **d**) were determined using real-time RT-PCR analysis. Shown is the mean ± SEM of *n* = 3 of experimental replicates from three different culture of CT- and DD-fibroblasts, each performed in triplicate. *p < 0.05, **p < 0.01

Since increased collagen accumulation is the hallmark for fibrosis and the progression of Dupuytren’s disease [33], our next goal was to investigate if PFD can inhibit collagen accumulation. Interestingly, we found that both basal expression and TGF-β_1_ induction of type I (α-2 chain; *COL1A2*) and type III collagen (α-1 chain; *COL3A1*) mRNA levels were significantly inhibited in both CT- and DD- derived fibroblasts by addition of PFD (Fig. 6a – d). PFD appears to more greatly inhibit collagen message in DD cells compared to CT cells. These fingings reiterate PFD as an attractive candidate to mitigate collagen accumulation (and therefore disease phenotype) in DD.

### PFD suppresses TGF-β_1_- induced fibronectin protein levels

**Fig. 7:**
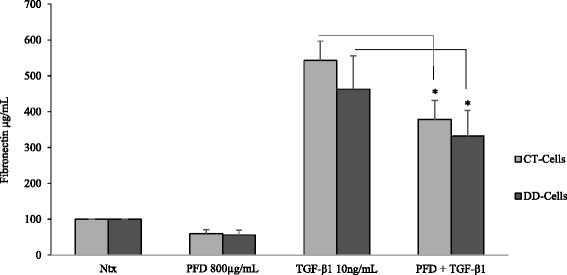
Pirfenidone substantially reduced TGF-β_1_ induction of fibronectin expression. CT- and DD-cord derived fibroblasts grown in growth medium overnight were treated with medium containing 0.1 % dialyzed FBS for 24 h. Following which, cells were treated with 800 μg/mL of PFD with or without 10 ng/ml TGF-β_1_ for 24 h. After the treatment, secreted proteins in the growth medium were collected to determine the fibronectin expression using Human Fibronectin SimpleStep ELISA Kit. CT- and DD-derived fibroblasts derived from three different patient samples (N = 3/group) were used to perform the experiment three times in triplicate. Statistical significance was determined by One-way ANOVA. *p < 0.05

The palmar fascia fibrosis seen in DD is associated with excessive deposition of extracellular matrix (ECM) components including fibronectin [19]. DD tissues and cells have been reported to show increased expression of fibronectin type III extra-domain B (*FN* 1-ED-B) and “oncofetal” fibronectin levels [34, 35] which prompted us to determine if PFD can play a role in reducing fibronectin levels. We found that PFD (800 μg/ml) decreased TGF-β_1_- induced fibronectin protein levels in both CT- and DD-derived fibroblasts (Fig. 7).

### PFD inhibits TGF-β_1_-induced phosphorylation of Smad2/Smad3

**Fig. 8:**
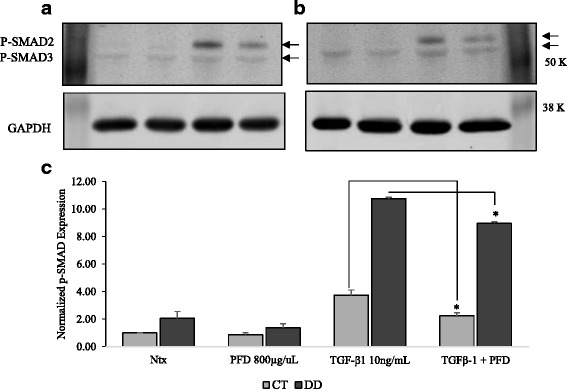
Pirfenidone inhibits TGF-β_1_-induced phosphorylation of Smad2/Smad3. CT-and DD-cord-derived fibroblasts derived from three different patient samples (N = 3/group) were maintained in MEM- α medium containing 0.1 % dialyzed FBS for 24 h. After 24 h, cells were either treated or untreated with PFD (800 μg/ml) in the presence or absence of TGF-β_1_ (10 ng/ml) for additional 24 h. Cell lysates collected from CT- (**a** & **b**) and DD- derived fibroblasts were used to examine the expression of phosphorylated Smad2/Smad3 by Western blot analysis. Proteins obtained from different patients were processed in parallel to confirm the changes that are observed are consistent. Densitometry results are reported as the ratio of phosphorylated Smad2/Smad3 protein to GAPDH expression (**c**). Shown here is the representation of Western blot experiments performed using three different culture each of CT- and DD-derived fibroblasts essentially with identical results. *p < 0.05

Studies have shown that *TGF-*β_1_ is a potent modulator of fibroblast and myofibroblast proliferation and differentiation [7]. All key components of the TGF-β_1_/Smad signaling cascade were noted to have increased expression patterns in DD, resulting in accelerated TGF-β_1_ signaling [36]. We examined the phosphorylation of Smad2/Smad3 protein as a measure for active canonical TGF-β_1_ signaling. We detected an increase in protein levels of phospho-Smad2/3 when stimulated with TGF-β_1_ in both CT- and DD-derived fibroblasts. This increase in expression was attenuated by the addition of PFD (Fig. 8a & b).

### Statistical analysis

Statistical analyses were performed by one way ANOVA using GraphPad Prism version 7.0. Statistically significant differences between paired experimental and control groups; and paired treatments between CT cells and DD cells was performed using Dunnett’s and Sidak’s analyses. One sample *T*-test was used to compare the background between CT and DD cell population. A *p* value <0.05 was considered significant.

## Discussion

DD is a chronic, fibroproliferative disorder with high recurrence rate after all available strategies for treatment. The cellular and molecular physiology underlying this trend to recurrence remains under investigation. Our work has shown that not only fibroblasts from actively diseased cords, but also fibroblasts from phenotypically normal adjacent palmar fascia in DD have significantly altered patterns of gene expression compared to control cells from CT patients [37]. These nearby cells may therefore represent a residual manifestation of proto-disease after surgery, which may be stimulated by cytokine mediators to progress to active, frank recurrence. An important such mediator, known to promote the conversion of fibroblasts to an activated myofibroblast phenotype and known to play a role in DD, is TGF-β_1_. Accordingly, therapies that can counteract TGF-β_1_ may also prove useful in dealing with DD.

The link between elevated TGF-β_1_ and fibrosis is well documented but continues to grow. A recent study by Meyer et al. [38] shows that increased circulating levels of plasma TGFβ_1_ released from platelets contribute to cardiac fibrosis that occurs in response to aortic constriction during cardiac surgery in humans. There is no study to date that correlates the presence of TGF-β_1_ in the palmar fascial tissues with elevated levels in circulating plasma in patients suffering from DD, but neither to our knowledge has such a study been attempted. Interestingly, a case study by Cutolo et al. [39] describes a case of symmetric bilateral posterior subcapsular cataracts associated with symmetric bilateral Dupuytren’s disease. In this study the authors found that the patient’s serum TGF-β_1_ concentration was almost double (18,290 pg/ml) compared to the cataract patients that did not have Dupuytren’s disease (9961.06 pg/ml). Such observations suggest that agents that will counteract TGF-β_1_ may be useful adjuncts in the treatment of DD, especially to guard against disease recurrence.

One such agent is Pirfenidone. Multiple in vitro and in vivo studies have been performed to show the anti-inflammatory and anti-fibrotic beneficial effects of PFD on cell populations derived from various tissue sources associated with fibrosis [29, 40–42]. Since PFD is already FDA-approved for use in pulmonary fibrosis, there is a substantial history of safety in human use. Our present study seeks to verify that administration of PFD can elicit similar anti-fibrotic effects against the altered physiology of Dupuytren’s disease at the cellular level. We find that PFD is able to inhibit cell proliferation, migration (after TGF-β_1_ stimulation), and contraction of DD fibroblasts, and also inhibits the expression of collagen and fibronectin, two key components of the DD ECM. A similar effect (sometimes not quite as pronounced) is seen in CT fibroblasts, as may be expected from an agent known to have broadly anti-fibrotic effects in a variety of tissue and disease circumstances. These observations indicate that PFD should be considered as a therapeutic agent against DD in a clinical trial. Others have likewise suggested the use of other anti-TGF-β_1_ medicaments such as N-acetyl-L-cysteine (NAC) and ACE inhibitors as therapeutic interventions for DD [43], but no such trial has, to our knowledge been carried out.

TGF- β_1_ can signal via the Smad signaling pathways or by the activation of non-Smad signaling pathways including MAP kinase, Rho GTPase, and PI3 kinase-Akt, resulting in repression of epithelial marker genes and activation of mesenchymal markers [44, 45]. In order to explore the mechanisms through which TGF- β_1_ actions are inhibited by PFD we examined the Smad-dependent pathways. Studies from other cell types indicate that PFD counteracts TGF- β_1_ action by down-regulating phosphorylation of Smad proteins [46–49]. A previous study by Krause et al., [36] shows that DD tissues have elevated basal expression of Smad2 and Smad3, and P-Smad2 levels were found to be elevated, but not P-Smad3 levels. In our DD- derived fibroblasts in culture we did not find any such increase in the basal phosphorylation of Smad2/Smad3 compared to CT cells, but posit this may be due to the lack of stimulation by TGF- β_1_ (whereas DD tissues in vivo may be expected to see such stimulation). When TGF-β_1_ was added to cells, we did see significantly increased expression of P-Smad2 but not P-Smad3 in DD- derived fibroblasts, consistent with the observation in tissues. CT-derived fibroblasts also showed this increase in culture, but would not be expected to in vivo, where presumably there is little TGF-β_1_ stimulation in CT disease. Importantly, in both cell types the increase in P-Smad2 was negated by the addition of PFD. This does suggest that, in both DD and control palmar fascial fibroblasts. PFD is acting at least partially through suppression of the canonical Smad signaling pathway.

In sum, we show that PFD has significant physiologic effects and anti-TGF-β_1_ properties in palmar fascial fibroblasts as it does in cells from other tissue sources. Importantly, PFD is effective against actively diseased fibroblasts from DD patients, and may therefore be expected to have a similar potency in vivo, potentially mitigating DD disease and recurrence. It remains to be determined how best to deliver PFD to the target cell populations in the setting of DD; to date its use has largely been as a systemic (oral) medication, which may also prove useful against DD. However, gel formulations of PFD intended for topical administration have also been described. A recent clinical trial by Armendariz-Borunda et al. [27] shows that topical administration of 8 % PFD gels is effective and safe in the treatment of hypertrophic scars caused by burns in children. Another recent study by Rodríguez-Castellanos et al. [50] shows that topical application of 8 % PFD gels to localized scleroderma reduces both inflammation and fibrosis. These studies raise the prospect of using PFD locally in DD patients rather than via systemic oral administration. The main impediment to such a scheme may be in delivering sufficient PFD to cross the skin barrier to underlying palmar fascial tissue. However, we anticipate that doses used in this study will be achievable in vivo. Since we are aiming for topical application of PFD for use in DD patients, we expect that we can achieve the effect of the drug locoregionally with much less systemic exposure than used for treating other fibrotic conditions such as idiopathic pulmonary fibrosis (IPF). Future studies will focus on optimizing strategies to effectively deliver PFD to the palmar fascia, and to verify its ability to inhibit DD disease phenotype using a recently described orthotopic animal model of DD [31].

## Conclusions

Taken together these results suggest the PFD can potentially prevent TGF-β_1_-induced fibroblast to myofibroblast transformation and inhibit ECM production mainly Type I- and Type III- collagen and fibronectin in DD-derived fibroblasts. Further in-vivo studies with PFD may lead to a novel therapeutic application in preventing the progression or recurrence of Dupuytren’s disease.

## Abbreviations

ANOVA: Analysis of variance
CT: Carpal tunnel
DD: Dupuytren’s disease
sFPCL: Stressed Fibroblast -Populated Collagen Lattice
TGF-β_1_: Transforming growth factor-beta
α – SMA: alpha smooth muscle actin
